# Supplementary material for: Ambient Air Pollution, Extreme Temperatures and Birth Outcomes: A Protocol for an Umbrella Review, Systematic Review and Meta-Analysis
Source: Int J Environ Res Public Health. 2020 Nov 21;17(22):8658. doi: 10.3390/ijerph17228658 (PMC7700558; doi:10.3390/ijerph17228658)
Supplement: Supplementary file 1 [file ijerph-17-08658-s001.pdf]

Table S1. Advanced literature search in Title/Abstract for PubMed.

Set #	Advanced Search Within the Title and Abstract with the Function 'Title/Abstract'
1	"air pollut*"[Title/Abstract] OR "particulate matter*"[Title/Abstract] OR "carbon monoxide"[Title/Abstract] OR "sulfur dioxide"[Title/Abstract] OR "sulphur dioxide"[Title/Abstract] OR "nitrogen dioxide"[Title/Abstract] OR "nitrogen oxides"[Title/Abstract] OR "nitric oxide"[Title/Abstract] OR ozone[Title/Abstract] OR "gaseous pollut*"[Title/Abstract] OR "fine partic*"[Title/Abstract] OR "air qualit*"[Title/Abstract] OR "total suspended partic*"[Title/Abstract] OR "PM10"[Title/Abstract] OR "PM2.5"[Title/Abstract] OR "NO2"[Title/Abstract] OR "SO2"[Title/Abstract] OR "NOx"[Title/Abstract] OR "CO"[Title/Abstract] OR "O3"[Title/Abstract] OR "TSP"[Title/Abstract] OR "temperature*"[Title/Abstract] OR weather*[Title/Abstract] OR heat*[Title/Abstract] OR cold*[Title/Abstract] OR "climat*"[Title/Abstract] OR "heat wave*"[Title/Abstract] OR heatwave*[Title/Abstract] OR "cold wave*"[Title/Abstract] OR coldwave*[Title/Abstract] OR "thermal stress"[Title/Abstract] ; Filters: English
2	"Pregnancy Outcome*"[Title/Abstract] OR "Birth Outcome*"[Title/Abstract] OR "Perinatal Outcome*"[Title/Abstract] OR "Obstetric Outcome*"[Title/Abstract] OR "Fetal Outcome*"[Title/Abstract] OR "Foetal Outcome*"[Title/Abstract] OR "Spontaneous Abortion"[Title/Abstract] OR "Premature Birth"[Title/Abstract] OR "Preterm Birth"[Title/Abstract] OR "Preterm Delivery"[Title/Abstract] OR "Premature Labo*"[Title/Abstract] OR Stillbirth[Title/Abstract] OR "Still birth"[Title/Abstract] OR "Fetal Death"[Title/Abstract] OR "Foetal Death"[Title/Abstract] OR "Pregnancy Loss"[Title/Abstract] OR Miscarriage[Title/Abstract] OR "Perinatal Death"[Title/Abstract] OR "Birth Weight"[Title/Abstract] OR "Birthweight"[Title/Abstract] OR "Fetal Weight"[Title/Abstract] OR "Foetal Weight"[Title/Abstract] OR "Fetal Growth"[Title/Abstract] OR "Foetal Growth"[Title/Abstract] OR "Gestational Age"[Title/Abstract] OR "Small-for-gestational age"[Title/Abstract] OR "intra-uterine growth retardation*"[Title/Abstract] OR "intrauterine growth retardation*"[Title/Abstract] OR "intrauterine growth restriction*"[Title/Abstract] OR "intra-uterine growth restriction*"[Title/Abstract] OR "PTB"[Title/Abstract] OR "PTD"[Title/Abstract] OR "LBW"[Title/Abstract] OR "TLBW"[Title/Abstract] OR "SGA"[Title/Abstract] OR "FGR"[Title/Abstract] OR "IUGR"[Title/Abstract] ; Filters-English
3	#1 AND 2
4	"review" OR "meta-analysis"[Title/Abstract]
5	# 3 AND #4
6	Afghanistan[Title/Abstract] OR "Guinea-Bissau"[Title/Abstract] OR "Sierra Leone"[Title/Abstract] OR "Burkina Faso"[Title/Abstract] OR Haiti[Title/Abstract] OR Somalia[Title/Abstract] OR Burundi[Title/Abstract] OR "North Korea"[Title/Abstract] OR "South Sudan"[Title/Abstract] OR "Central African Republic"[Title/Abstract] OR Liberia[Title/Abstract] OR Sudan[Title/Abstract] OR Chad[Title/Abstract] OR Madagascar[Title/Abstract] OR Syria[Title/Abstract] OR "Syrian Arab Republic"[Title/Abstract] OR Congo[Title/Abstract] OR Malawi[Title/Abstract] OR Tajikistan[Title/Abstract] OR Eritrea[Title/Abstract] OR Mali[Title/Abstract] OR Togo[Title/Abstract] OR Ethiopia[Title/Abstract] OR Mozambique[Title/Abstract] OR Uganda[Title/Abstract] OR Gambia[Title/Abstract] OR Niger[Title/Abstract] OR Yemen[Title/Abstract] OR Guinea[Title/Abstract] OR Rwanda[Title/Abstract] OR Angola[Title/Abstract] OR Honduras[Title/Abstract] OR "Papua New Guinea"[Title/Abstract] OR Algeria[Title/Abstract] OR India[Title/Abstract] OR Philippines[Title/Abstract] OR Bangladesh[Title/Abstract] OR Kenya[Title/Abstract] OR "São Tomé"[Title/Abstract] OR Principe[Title/Abstract] OR Benin[Title/Abstract] OR Kiribati[Title/Abstract] OR Senegal[Title/Abstract] OR Bhutan[Title/Abstract] OR "Kyrgyz Republic"[Title/Abstract] OR "Solomon Islands"[Title/Abstract] OR Bolivia[Title/Abstract] OR "Lao PDR"[Title/Abstract] OR "Sri Lanka"[Title/Abstract] OR "Cabo Verde"[Title/Abstract] OR Lesotho[Title/Abstract] OR Tanzania[Title/Abstract] OR Cambodia[Title/Abstract] OR Mauritania[Title/Abstract] OR "Timor-Leste"[Title/Abstract] OR Cameroon[Title/Abstract] OR Micronesia[Title/Abstract] OR Tunisia[Title/Abstract] OR Comoros[Title/Abstract] OR Moldova[Title/Abstract] OR Ukraine[Title/Abstract] OR Congo[Title/Abstract] OR Mongolia[Title/Abstract] OR Uzbekistan[Title/Abstract] OR "Côte d'Ivoire"[Title/Abstract] OR Morocco[Title/Abstract] OR Vanuatu[Title/Abstract] OR Djibouti[Title/Abstract] OR Myanmar[Title/Abstract] OR Vietnam[Title/Abstract] OR Egypt[Title/Abstract] OR Nepal[Title/Abstract] OR "West Bank"[Title/Abstract] OR Gaza[Title/Abstract] OR "El Salvador"[Title/Abstract] OR Nicaragua[Title/Abstract] OR Zambia[Title/Abstract] OR Eswatini[Title/Abstract] OR Nigeria[Title/Abstract] OR Zimbabwe[Title/Abstract] OR Ghana[Title/Abstract] OR Pakistan[Title/Abstract] OR Albania[Title/Abstract] OR Fiji[Title/Abstract] OR Montenegro[Title/Abstract] OR "American Samoa"[Title/Abstract] OR Gabon[Title/Abstract] OR Namibia[Title/Abstract] OR Argentina[Title/Abstract] OR Georgia[Title/Abstract] OR "North Macedonia"[Title/Abstract] OR Armenia[Title/Abstract] OR Grenada[Title/Abstract] OR Paraguay[Title/Abstract] OR Azerbaijan[Title/Abstract] OR Guatemala[Title/Abstract] OR Peru[Title/Abstract] OR Belarus[Title/Abstract] OR Guyana[Title/Abstract] OR Russia[Title/Abstract] OR Belize[Title/Abstract] OR Indonesia[Title/Abstract] OR Samoa[Title/Abstract] OR Bosnia[Title/Abstract] OR Herzegovina[Title/Abstract] OR Iran[Title/Abstract] OR Serbia[Title/Abstract] OR Botswana[Title/Abstract] OR Iraq[Title/Abstract] OR "South Africa"[Title/Abstract] OR Brazil[Title/Abstract] OR Jamaica[Title/Abstract] OR "Saint Lucia"[Title/Abstract] OR Bulgaria[Title/Abstract] OR Jordan[Title/Abstract] OR "Saint Vincent"[Title/Abstract] OR Grenadines[Title/Abstract] OR China[Title/Abstract] OR Kazakhstan[Title/Abstract] OR Suriname[Title/Abstract] OR Colombia[Title/Abstract] OR Kosovo[Title/Abstract] OR Thailand[Title/Abstract] OR "Costa Rica"[Title/Abstract] OR Lebanon[Title/Abstract] OR Tonga[Title/Abstract] OR Cuba[Title/Abstract] OR Libya[Title/Abstract] OR Turkey[Title/Abstract] OR Dominica[Title/Abstract] OR Malaysia[Title/Abstract] OR Turkmenistan[Title/Abstract] OR "Dominican Republic"[Title/Abstract] OR Maldives[Title/Abstract] OR Tuvalu[Title/Abstract] OR "Equatorial Guinea"[Title/Abstract] OR "Marshall Islands"[Title/Abstract] OR Venezuela[Title/Abstract] OR Ecuador[Title/Abstract] OR Mexico[Title/Abstract] OR

	"developing countries"[Title/Abstract] OR "low-to middle-income countries"[Title/Abstract] OR "low-income countries"[Title/Abstract] OR "middle-income countries"[Title/Abstract] OR "lower-middle-income countries"[Title/Abstract] OR "upper-middle-income countries"[Title/Abstract] OR "LMICs"[Title/Abstract] OR Africa*[Title/Abstract] OR Asia*[Title/Abstract] OR "Latin America*" [Title/Abstract] OR Caribbea*[Title/Abstract] OR "Middle East"[Title/Abstract] OR "Pacific*"[Title/Abstract]
7	#3 AND #6

Table S2. Risk of bias appraisal checklist for environmental health observational studies (modified from [1] with information from [2–4]).

Bias Criteria Item	Indicators and Quality Scoring *
1 Publication type	0 = Not peer reviewed<br>1= Peer reviewed
2. Study design	0 = Ecological<br>1 = Cross-sectional<br>2 = Case control<br>3 = Cohort, retrospective<br>4 = Cohort, prospective
3. Selection of participants	0 = No information provided<br>1 = No random sampling OR response rate less than 60% OR attrition rate higher than 20% OR purposeful sampling OR sampling from different population with inconsistent inclusion/exclusion criteria, different time frame, different populations with heterogenous proportion in each group.<br>3 = Participants randomly sampled from a known/same population at same time frame AND response rate higher than 60%/whole source population sampled AND attrition rate less than 20% in follow-up studies OR consistent exclusion/inclusion criteria to recruit participants from the same population at the same time frame OR from different populations with uniform proportion in each group OR participant selection is not an element of study design (ecological study where whole population of interest is sourced)
4. Assessment of exposure (air pollution or meteorological factors)	0 = Subjective method (e.g., self-report by mother, using proxies)<br>1 = Objective method, low accuracy (semi-individual level by proximity to ground monitors or regional level exposure assessments by using average exposure in a region or a grid with low spatial resolution or modelling with no ground-based monitoring data) OR surrogate as distance to source<br>2 = Objective method, limited accuracy or validity (e.g., individual or postcode/street-level exposure by complex modelling such as land-use regression, dispersion models, geostatistical interpolation, hybrid models, personal air sampling models etc with ground-based monitoring data and land-use variables)<br>3 = Objective method, accurate and valid (e.g. individual or postcode/street-level exposure by the complex models with ground-based monitoring data, land-use variables and satellite derived aerosol data<br>4 = Objective method, highly accurate and valid (e.g., prospective direct personal real-time activity measurement using portable personal sensors or exposomic technologies OR validated modelling that accounted for the real time-activity pattern of each participant and with spatial accuracy)
5. Time frame of exposure (Air pollution or meteorological)	0 = Timeframe outside pregnancy and delivery<br>1 = Exposure during short period OR at the date of delivery<br>3 = Exposure during entire pregnancy period
6. Assessment of birth outcomes	0 = Subjective assessment by report of mother<br>2 = Objective (e.g., from medical records)
7. Confounding factors	0 = None OR group-level OR only one important confounding factor considered (e.g., maternal age or tobacco/alcohol use or education/socioeconomic position or race/ethnicity, parity/gravidity, sex of neonate)<br>1 = Confounding factors considered for at least two of the following: maternal age, tobacco/alcohol use, education/socioeconomic position, parity/gravidity, sex of neonate, gestational age (for birth weight and LBW—not relevant for term births)<br>2 = Consideration of all of the above confounders<br>3 = Consideration of all of the above and season of birth/conception or any other air pollutant/climatic factor<br>4 = Consideration of all of the above and at least one of the following: race/ethnicity, marital status, obstetric history/comorbidity, maternal body mass index, urbanicity
8. Missing values	0 = No statement on absence or handling missing values described

	1= Statement on missing values and insufficiently described<br>2= Statement on missing values and handling missing values was sufficiently described but reasons likely to be related to true outcome, with either imbalance in numbers or reasons for missing data across exposure groups OR Potentially inappropriate application of imputation.<br>3= Clear statement of no missing values OR Handling missing values was sufficiently described and with no/little likelihood of affecting exposure/outcome
9. Statistical analysis	0 = Flaws in OR inappropriate statistical testing OR interpretation of statistical tests that may have affected result<br>1= Appropriate statistical testing but biased interpretation of results<br>2 = Appropriate statistical testing and interpretation of tests without bias
10. Reporting of research results	0 = Not all of the study's pre-specified primary outcomes in the protocol or methods, abstract, and/or introduction have been reported OR one or more primary outcomes is reported that were not pre-specified OR one or more reported primary outcomes were not pre-specified (except for an unexpected effect) OR one or more outcomes of interest are reported incompletely OR there is insufficient information about selective outcome reporting but there is indirect evidence suggesting that the study was not free of selective reporting<br>1= There is insufficient information about selective outcome reporting to permit a judgment of low risk of bias, but there is indirect evidence suggesting that the study was free of selective reporting<br>2 = All of the study's pre-specified (primary and secondary) outcomes outlined in the protocol, methods, abstract, and/or introduction that are of interest in the review have been reported in the pre-specified way OR there is evidence that selective outcome reporting is not capable of introducing risk of bias in the study
11. Limitations	0 = No OR insufficient/unclear description of limitations<br>1= Described the limitations of the study, considering sources, direction and magnitude of potential bias or imprecision.
12. Funding and Conflict of Interest	0 = No statement on funding OR/AND Conflict of interest<br>1= Gave statement on conflict of interest OR/AND source of funding and/or the role of the funders for the present study and/or for the original study on which the present article is based and there is Potential conflict of interest or financial gain from the funder and/role of the funder<br>2 = Gave statement on conflict of interest OR/AND source of funding and/or the role of the funders for the present study and/or for the original study on which the present article is based and there is No Potential conflict of interest or financial gain from the funder and/role of the funder
13. Additional bias	0 = Several other study design or conduct issues that may have led to bias<br>1 = One other serious study flaw<br>2 = No other study serious flaws

* Possible total score = 33; 26–33 (i.e. at least 80% of maximum score 33) is low RoB, 17–25 (i.e. less than 26 but ≥ 50% of max 33) is moderate RoB, and <17 is high RoB.

Table S3. Data extraction tool for the systematic review 1 (umbrella review).

**Domain**
First author (Year)
Review type (SR, MA, SRMA)
Aims/Objectives
Databases searched (number, names, dates searched, and language restriction)
Grey/non-databases searched or No
Review guidelines used or No
Number of Primary studies included and no. of each study design
Publication date range of primary studies
Total participants included in the review
Description of participants in the review
Exposure assessments
Outcomes assessments
RoB tools used or No
Quality ratings of primary studies or No
Method of data synthesis
Overall results for non-quantitative synthesis

Meta-analytic results (effect estimates, CIs, p-value)
Heterogeneity and publication tests and results
Results of main subgroup/sensitivity analyses
Main researchers' in-text summary statement/conclusion
Main findings and interpretations in abstract
Researchers' recommendations
Researchers' stated strengths and limitations
Reported on funding sources (yes/no) and role of funder (yes/no); Conflict (yes/no)
Review protocol registered (yes/no)? published (yes/no)? for yes, where?
If No for both previous items, any evidence of pre-specified review method? (Yes, specify; No)
No. of citations (from Google Scholar)

Abbreviations: SR-systematic review, MA- meta-analysis, SRMA- systematic review and meta-analysis, RoB- risk of bias.

Table S4. Data extraction tool for the systematic review 2 (systematic review and meta-analysis in LMICs).

**Title**
First author (Year)
Journal
Study setting (country, state, region)
Birth data source, study population and sample size (case and control)
Sampling period and procedure
Study design
Birth outcome (definitions, assessment, rate)
Exposure (source, assessment method and timeframe)
Statistical methods
Exposure levels (mean, median, quartiles/percentiles, range)
Main statistical findings (crude and adjusted effect estimates and reference unit with 95% confidence intervals and p-values); entire, trimester, other timeframes reported
Adjustment of confounding factors
Subgroup/sensitivity analysis
Study country's World Bank economic group *
Study country's Global Gender Gap Index *
Study country's Global Hunger Index *
Study country's Political Stability Index *
Study country's climatic zone (tropical, subtropical, temperate, polar/cold) *

* These extra-study data will be extracted from the respective data sources.

Table S5. Grading the importance of an exposure (adapted from [5]).

**Category of Importance of Exposure**	**Grading Criteria**
++	The exposure variable has been found to be positively associated with the birth outcome in all reviews, without exception. This could mean that only one review has included a particular exposure and showed that this was a significant positive association and/or reported a (non)-significant large effect size of RR * > 1.50. But it could also mean that a number of reviews were conducted that included this exposure and birth outcome, and

	all of them concluded that the exposure was significantly positively related to a particular birth outcome.
+	This implies that not all but ≥ 80% of the available reviews concluded the exposure to be positively related to the birth outcome, or the separate reviews reported that 80% or more of the original studies concluded the factor to be positively related. This could therefore mean that only one review has included a particular exposure and birth outcome and showed that this was a significant positive association in ≥ 80% of the primary studies. But it could also mean that a number of reviews were executed towards this exposure and most (≥80%), but not all, concluded that the exposure was positively related to the birth outcome.
0	The exposure has been found to be related and/or reported a (non)-significant large effect size of RR >1.50 in some reviews (20% to 80% of available reviews or of the primary studies reviewed in these reviews) but not in others with mixed findings. The direction of the association can be either positive or negative or null hypothesis. This could mean that only one review has included a particular exposure and outcome and showed \`mixed findings' in 20% to 80% of the included primary studies. But it could also mean that results are mixed across 20% to 80% reviews.
00	The exposure was found to have no association with the birth outcome (a null hypothesis) in all reviews, without exception.
-	This implies that not all but ≥ 80% of the available reviews concluded the exposure to be negatively related to the birth outcome, or the separate reviews report that 80% or more of the original studies concluded the factor to be negatively related. This could therefore mean that only one review has included a particular exposure and birth outcome and showed that this was a significant negative association in ≥ 80% of the included primary studies. But it could also mean that a number of reviews were executed towards this exposure and most (≥ 80%) but not all, concluded that the exposure was negatively related to the birth outcome.
- -	The exposure has been found to be negatively associated in all reviews, without exception. This could mean that only one review has included a particular variable and showed that this was a significant negative correlate and/or reported a (non)-significant large effect size of RR > 1.50. But it could also mean that a number of reviews were conducted that included this exposure and all of them concluded that the exposure was negatively related to the particular birth outcome.

* With no available empirically defined "large magnitude of effect" and instead of making decision on 'large magnitude of effect' on project-specific basis for reviews in evaluating quality of evidence of studies on the association between a certain environmental risk factor and health outcomes, WHO environmental noise guidelines for the European Region, chose RR of at least 1.50 with the believe that it is highly unlikely that an RR ≥ 1.50 can be explained by residual confounding in environmental epidemiology after adjusting for other major risk factors [6].

Table S6. Grading the strength of evidence (adapted from [5]).

Category of Strength of Evidence	Grading Criteria
Convincing evidence (Ce)	Evidence based on studies showing consistent associations between the exposure and the birth outcome. The available evidence is based on a substantial number of high-quality studies, including longitudinal observational studies and where relevant, experimental studies of sufficient sample size, duration and quality showing consistent effects. Specifically, the grading criteria include evidence from more than one study type and evidence from at least two independent cohort studies should be available, and strong and plausible experimental evidence.

Probable evidence (Pe)	Evidence based on studies showing fairly consistent associations between the exposure and the birth outcome, but there are shortcomings in the available evidence or some evidence to the contrary, which precludes a more definite judgment. Shortcomings in the evidence may be any of the following: inconsistency of the results, insufficient studies available (but evidence from at least two independent cohort studies or five case-control studies should be available), inadequate sample sizes, insufficient duration of studies, incomplete follow-up.
Limited, suggestive evidence (Lse)	Evidence based mainly on findings from cross sectional studies. Insufficient longitudinal observational studies or experimental studies are available, or results are inconsistent. More well-designed high-quality studies are required to support the tentative exposure-outcome associations.
Limited, no conclusive evidence (Lnce)	Evidence based on findings of a few studies which are suggestive but are insufficient to establish an association between the exposure and the birth outcome. Evidence based solely on cross-sectional studies, no evidence available from longitudinal observational or experimental studies. More well-designed high-quality studies are required to support the tentative exposure-outcome associations.

Table S7. Strength of evidence of plausible toxicological effects of exposures on birth outcomes (adapted from [3,4,7]) #.

**Strength of Rating**	**Definition**
Sufficient evidence of toxicity	A positive relationship is observed between exposure and outcome where chance, bias, and confounding can be ruled out with reasonable confidence. The available evidence includes results from one or more well-designed, well-conducted studies, and the conclusion is unlikely to be strongly affected by the results of future studies.
Limited evidence of toxicity	A positive relationship is observed between exposure and outcome where chance, bias, and confounding cannot be ruled out with reasonable confidence. Confidence in the relationship is constrained by such factors as: the number, size, or quality of individual studies or inconsistency of findings across individual studies. As more information becomes available, the observed effect could change, and this change may be large enough to alter the conclusion.
Inadequate evidence of toxicity	The available evidence is insufficient to assess effects of the exposure. Evidence is insufficient because of the limited number or size of studies, low quality of individual studies, or inconsistency of findings across individual studies. More information may allow an assessment of effects.
Evidence of lack of toxicity	No relationship is observed between exposure and outcome, and chance, bias and confounding can be ruled out with reasonable confidence. The available evidence includes consistent results from more than one well-designed, well-conducted study at the full range of exposure levels that humans are known to encounter, and the conclusion is unlikely to be strongly affected by the results of future studies. The conclusion is limited to the age at exposure and/or other conditions and levels of exposure studied.

Grading the strength of evidence is based on combination of four criteria: (i) Quality of overall body of evidence (ii) Direction of effect (iii) Confidence in effect (likelihood that a new study will change our conclusion) and (iii) Other compelling attributes of the data that may influence certainty [3,5,7].

- Reflection of PECO components in the research question and/or inclusion criteria (item 1)
- Protocol registered or/and published protocol before commencement of the review (item 2)
- Adequacy of the literature search (item 4)
- Justification for excluding individual studies (item 7)
- Risk of bias from individual studies being included in the review (item 9)
- Appropriateness of meta-analytical methods (item 11) *
- Consideration of risk of bias when interpreting the results of the review (item 13)
- Assessment of presence and likely impact of publication bias (item 15) *

* Items 11 and 15 are not applicable if meta-analysis was not performed

[illegible]

- **High**

• N o or one non-critical weakness: the systematic review provides an accurate and comprehensive summary of the results of the available studies that address the question of interest

- **Moderate**

• More than one non-critical weakness *: the systematic review has more than one weakness but no critical flaws. It may provide an accurate summary of the results of the available studies that were included in the review

- **Low**

• One critical flaw with or without non-critical weaknesses: the review has a critical flaw and may not provide an accurate and comprehensive summary of the available studies that address the question of interest

- **Critically low**

• More than one critical flaw with or without non-critical weaknesses: the review has more than one critical flaw and should not be relied on to provide an accurate and comprehensive summary of the available studies

* Multiple non-critical weaknesses may diminish confidence in the review and it may be appropriate to move the overall appraisal down from moderate to low confidence.

Box S2. Rating overall confidence in the results of the existing review [8].

References

1. Dzhambov, A.M.; Lercher, P. Road Traffic Noise Exposure and Birth Outcomes: An Updated Systematic Review and Meta-Analysis. *Int. J. Environ. Res. Public Health* **2019**, *16*, 2522.
2. Stroup, D.F.; Berlin, J.A.; Morton, S.C.; Olkin, I.; Williamson, G.D.; Rennie, D.; Moher, D.; Becker, B.J.; Sipe, T.A.; Thacker, S.B.; et al. Meta-analysis of observational studies in epidemiology: A proposal for reporting. Meta-analysis Of Observational Studies in Epidemiology (MOOSE) group. *JAMA* **2000**, *283*, 2008–2012.
3. Chiu, W.; Johnson, N.; Moriarty, M.; Pulczinski, J.; Uwak, I.; Taiwo, S.; Lam, J.; Taylor, B.; Xu, X. Applying the Navigation Guide Systematic Review Methodology: Case Study #7. Association between Prenatal Exposures to Ambient Air Pollution and Birthweight. A Systematic Review of the Evidence Protocol. 2017. Available online: https://www.crd.york.ac.uk/PROSPEROFILES/58805_PROTOCOL_20170225.pdf (accessed on 13 June 2020).

4. Lam, J.; Sutton, P.; Kalkbrenner, A.; Windham, G.; Halladay, A.; Koustas, E.; Lawler, C.; Davidson, L.; Daniels, N.; Newschaffer, C.; et al. A Systematic Review and Meta-Analysis of Multiple Airborne Pollutants and Autism Spectrum Disorder. *PLoS ONE* **2016**, *11*, e0161851.
5. O'Donoghue, G.; Kennedy, A.; Puggina, A.; Aleksovska, K.; Buck, C.; Burns, C.; Cardon, G.; Carlin, A.; Ciarapica, D.; Colotto, M.; et al. Socio-economic determinants of physical activity across the life course: A "DEterminants of DIet and Physical ACtivity" (DEDIPAC) umbrella literature review. *PLoS ONE* **2018**, *13*, e0190737.
6. van Kempen, E.E.M.M.; Casas, M.; Pershagen, G.; Foraster, M. *Cardiovascular and Metabolic Effects of Environmental Noise: Systematic Evidence Review in the Framework of the Development of the WHO Environmental Noise Guidelines for the European Region*; Report No.: RIVM Report 2017-0078; National Institute for Public Health and the Environment: Utrecht, The Netherlands, 2017.
7. Woodruff, T.J.; Sutton, P. The Navigation Guide systematic review methodology: A rigorous and transparent method for translating environmental health science into better health outcomes. *Environ. Health Perspect*. **2014**, *122*, 1007–1014.
8. Shea, B.J.; Reeves, B.C.; Wells, G.; Thuku, M.; Hamel, C.; Moran, J.; Moher, D.; Tugwell, P.; Welch, V.; Kristjansson, E.; et al. AMSTAR 2: A critical appraisal tool for systematic reviews that include randomised or non-randomised studies of healthcare interventions, or both. *BMJ* **2017**, *358*, j4008.
